# Association of baseline core volume and early midline shift in acute stroke patients with a large ischaemic core

**DOI:** 10.3389/fneur.2022.1077824

**Published:** 2023-01-09

**Authors:** Ting-yu Yi, Yan-min Wu, Ding-lai Lin, Feng-long Lang, Yu-yan Yang, Zhi-nan Pan, Xiu-fen Zheng, Gan-ji Hong, Mei-hua Wu, Xiao-hui Lin, Rong-cheng Chen, Lisan Zeng, Wen-huo Chen, Yi Sui

**Affiliations:** ^1^Zhangzhou Affiliated Hospital of Fujian Medical University, Fujian, China; ^2^Department of Neurology, Fushun Central Hospital, Fushun, Liaoning, China; ^3^Department of Neurology, Shenyang First People's Hospital, Shenyang Medical College, Shenyang, China

**Keywords:** midline shift, large ischaemic core, prognosis, acute ischemic stroke, thrombectomy

## Abstract

**Background:**

Midline shift (MLS) is troublesome problem that may occur in patients with a large infarct core (LIC) and may be related to the baseline infarct core volume. The purpose of this study was to explore the relationship between baseline infarct core volume and early MLS presence.

**Materials and methods:**

Patients with acute intracranial large artery occlusion and a pretreatment relative cerebral blood flow (rCBF) <30% volume ≥50 ml on CT perfusion (CTP) were included, clinical outcomes following endovascular treatment (EVT) were retrospectively analyzed. The primary endpoint was MLS within 48 h (early MLS presence). The association between baseline ICV and early MLS presence was evaluated with multivariable regression.

**Results:**

Ultimately, 95 patients were included, and 29.5% (28/95) of the patients had early MLS. The number of patients with a baseline rCBF < 15% volume (median [interquartile range], 46 [32–60] vs. 29 [19–40]; *P* < 0.001) was significantly larger in the early severe MLS presence group. A baseline rCBF < 15% volume showed significantly better predictive accuracy for early MLS presence than an rCBF < 30% volume (area under the curve, 0.74 vs. 0.64, *P* = 0.0023). In addition, an rCBF < 15% volume ≥40 ml (odds ratio, 4.34 [95% CI, 1.571–11.996]) was associated with early MLS presence after adjustment for sex, age, baseline National Institutes of Health Stroke Scale score, onset-to-recanalization time.

**Conclusion:**

In patients with an acute LIC following EVT, a pretreatment infarct core volume > 40 ml based on an rCBF < 15% showed good predictive value for early MLS occurrence.

## Introduction

Endovascular thrombectomy (EVT) is the standard treatment for ischaemic stroke patients with large vessel occlusion (LVO) (1–8). However, most clinical trials of EVT focused on patients with small ischaemic cores defined as a volume with a relative cerebral blood flow (rCBF) <30% of <50 or <70 cm^3^ on computed tomographic perfusion (CTP) images or an Alberta Stroke Program Early CT Score [ASPECTS] ≥6 on non-contrast computed tomography (CT) imaging prior to thrombectomy (9). Recently, a meta-analysis showed that patients with a large ischaemic core (LIC) defined by an ASPECTS < 6, ischaemic core volume ≥ 50 ml or both can benefit from EVT (10). Moreover, a recent randomized clinical trial (RCT) performed in Japan showed that patients with an LIC had better functional outcomes with endovascular therapy than with medical care alone but had more intracranial hemorrhages (11).

However, one concern of patients with an LIC who receive EVT is cerebral oedema, which can lead to early cerebral midline shift (MLS) occurrence and poor prognosis. The causes of cerebral oedema remain unclear. Studies have shown that in patients with a core volume > 130 mL or CT-ASPECTS ≤ 3, EVT is not significantly associated with a clinical benefit and may be detrimental through the exacerbation of oedema. Therefore, we hypothesize that the baseline ischaemic core volume is associated with MLS occurrence.

Traditionally, the infarct core is defined as an rCBF < 30% (12); however, the chronological progression to infarction is different for different cell types (neurons are already severely damaged at a time and place where astrocytes are only minimally injured), and it is variable in both time and space (13). Currently, there is no optimal ischaemic core CTP parameter, and an rCBF < 30% ischaemic volume may also be reversible after reperfusion therapy, similar to the theory of diffusion MR reversibility (14, 15). Furthermore, we hypothesized that an rCBF < 15% ischaemic volume is extremely ischaemic tissue and is very difficult to save despite rapid reperfusion. In our study, we aimed to explore the relationship between an rCBF < 15% ischaemic volume and early MLS occurrence and determine the optimal ischaemic volume for predicting early MLS occurrence.

## Methods

### Patient population and data collection

The patient data analyzed in the present study were derived from our Prospective Registry database of Endovascular Treatment. For the present analysis, all patients enrolled between January 1, 2015, and July 1, 2021, were included. The inclusion criteria of this study were as follows: at least 18 years of age; acute occlusion of anterior intracranial large vessels and EVT; a baseline National Institutes of Health Stroke Scale (NIHSS) score ≥ 6; a baseline CTP and pretreatment ischaemic core volume ≥ 50 ml (autoassessed by CTP); a premorbid modified Rankin Scale (mRS) score ≤ 2; and an onset-to-presentation time within 24 h.

### Ethics approval

The study was approved by the local hospital ethics committees (ID 2021 LWB251). Written informed consent for EVT was obtained from the patient or the proxy. Informed consent for this study was waived because it was a retrospective study.

### Clinical variables and outcomes

The baseline data included sex, age, comorbidities (hypertension, diabetes, hyperlipidaemia, and atrial fibrillation), occlusion site, time from onset to treatment, neurologic deficits using the NIHSS score, and stroke subtype according to the Trial of ORG 10172 in Acute Stroke Treatment (TOAST) categories (16).

The primary outcome was midline shift (MLS), Secondary outcomes were functional independence at 3 months, corresponding to a modified Rankin Scale (mRS) score of 0–2, death at 3 months and symptomatic intracranial hemorrhage (sICH) according to the definition of Heidelberg (17).

### Radiological variables and outcomes

For the patients in our study, we used the following acquisition protocol and post-processing algorithms. CT perfusion was performed on a 320-slice scanner (GE Revolution). With each time point acquisition, a total of 320 slices with a thickness of 0.5 mm were obtained, which covered the whole brain (220 mm total coverage). Typically, 19 time points were obtained commencing 4 seconds after non-ionic iodinated contrast injection into an antecubital vein (50 mL, 5 mL/s; Bayer HealthCare). The acquisition parameters were 80 kilovolts (peak; kVp) and 200 mA. This acquisition also allowed the generation of intracranial angiographic data as well as perfusion maps. Whole-brain non-contrast CT (NCCT) was performed before CTP. After acquisition, CTP data were processed by the commercial software Mistar (Apollo Medical Imaging Technology). The mathematical model of delay-corrected singular value decomposition (dSVD) was chosen to generate perfusion parameters, which were presented as cerebral blood volume (CBV), cerebral blood flow (CBF), mean transit time (MTT), and delay time (DT). We further generated a penumbra/core map by setting thresholds for parametric maps. A DT > 3 sec was defined as hypoperfusion of brain tissue, and the ischaemic core volume was defined as rCBF < 30% (18). The mismatch volume (volume of DT > 3 s minus volume of rCBF < 30%) and mismatch ratio (volume of DT > 3 s divided by volume of rCBF < 30%) were also calculated by Mistar software (12). We can also derive the value of rCBF < 15%. The reperfusion grade was assessed with the Extended Thrombolysis in Cerebral Infarction (eTICI) scale (19), and substantial reperfusion was defined as a score of 2b, 2c, or 3 (19).

Early severe MLS presence was defined as an MLS greater than 3 mm (20) within 48 h post-EVT. MLS measurements were performed by readers who were blinded to the clinical data (Dr P.Z.N), and MLS was assessed as dichotomous (present or absent). In patients who had >1 follow-up scan, the scan with the highest MLS was analyzed. MLS was measured in millimeters at the level of the septum pellucidum (21, 22). The quantitative assessment of MLS was performed on patients previously categorized as having MLS. MLS measurements were independently assessed in 30% of the study cohort (29 consecutive subjects) by a second neurologist (Dr L.X.H) and found to have excellent interrater agreement (K = 0.95). Then, the patients were divided into 2 groups: the early severe MLS presence group was defined by the presence of an MLS > 3 mm, and the no early severe MLS presence group was defined by the presence of an MLS < 3 mm or MLS absence.

### Statistical analysis

Standard descriptive statistics are reported as medians and IQRs for continuous variables and as numbers and percentages for categorical variables. For between-group comparisons of categorical variables, the χ*2*-test or Fisher's exact test was used, as appropriate. The Mann–Whitney U test was employed for continuous variables. The areas under the curve from receiver operating characteristic (ROC) analyses were used to assess the predictive value of the baseline ischaemic core calculated by different levels of rCBF for early MLS presence. The Youden index was used to calculate the optimal cut-off predictive value of different rCBF threshold values. A bootstrap test was used to compare the areas under the curve (AUCs) between rCBF < 15% volume and rCBF < 30% volume. Multivariable logistic regression analyses were performed to determine the associations between early MLS presence and some important baseline factors, including core volume calculated by rCBF < 15%. Statistical analyses were performed using SPSS 18.0 (IBM SPSS, Chicago, IL) and R software version 4.13. A 2-sided *P* value < 0.05 was considered indicative of statistical significance.

## Results

**Figure 1:**
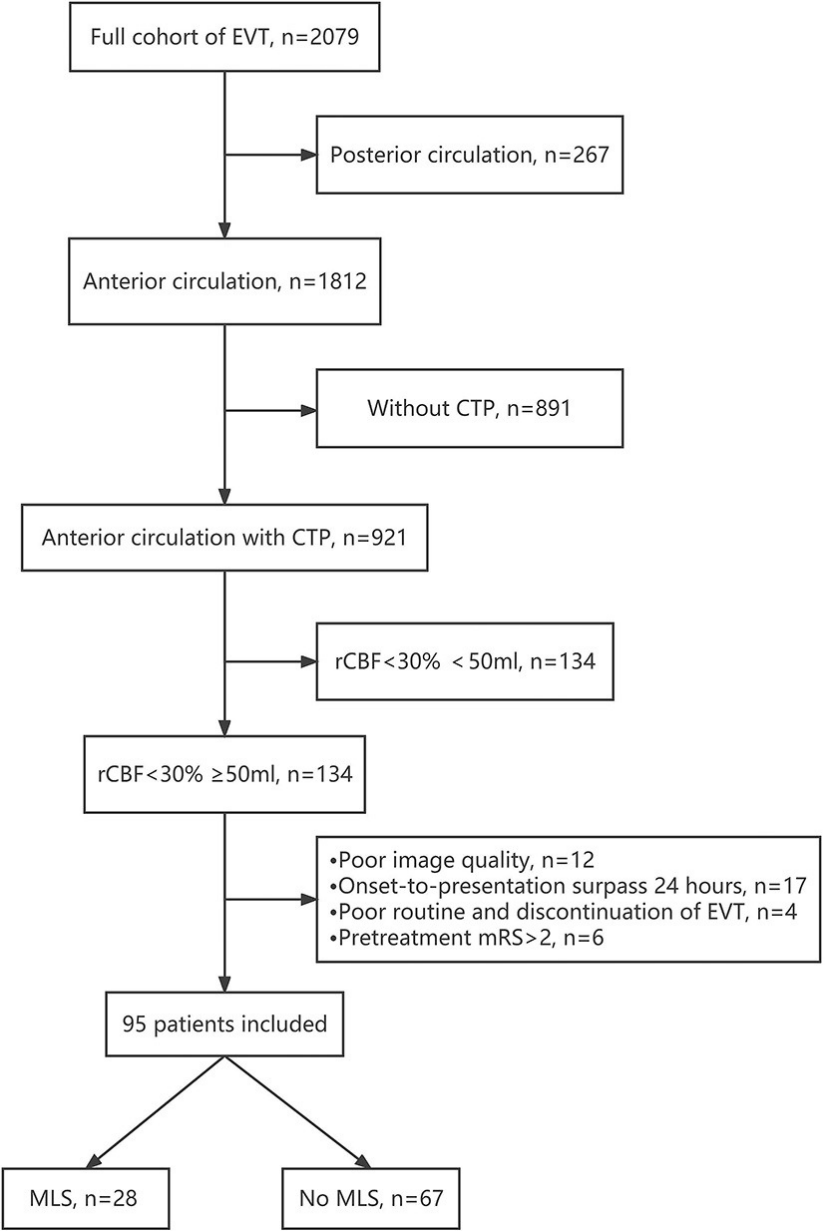
Study flow chart. CTP, CT perfusion; EVT, endovascular Therapy; MLS, midline shift.

The study flow chart is shown in Figure 1. Of the 2,079 patients enrolled in our registry database, 44.3% (921/2,078) of anterior circulation infarction patients had baseline CTP data, and 134 patients had a baseline core infarct volume ≥50 ml. Ultimately, 95 patients were included after the exclusion of 39 patients for the following reasons: (1) poor image quality (12 patients); (2) onset-to-presentation time surpassing 24 h (17 patients); (3) poor routine and discontinuation of endovascular therapy (4 patients); and (4) a pretreatment mRS > 2 (6 patients). A total of 29.5% (28/95) of patients had early severe MLS.

### Baseline characteristics and clinical outcomes of the whole cohort

Of the 95 included patients (Table 1), 58.9% (56/95) were males, and the median age was 72 (IQR, 63–79) years. The median baseline NIHSS score of this cohort was 19 (IQR, 16–22). A total of 49.5% (47/95) of the occlusion sites were intracranial carotid artery. Overall, successful revascularization (eTICI 2b,2c,3) was achieved in 98.9% (94/95) patients, and functional independence was achieved in 22.1% (21/95) of patients; 33.7% (31/95) of patients died.

**Table 1:** Comparison of baseline characteristics between patients with early severe MLS presence and without early severe MLS presence.

**Parameter**	**All patients *N =* 95**	**No MLS *N =* 67**	**MLS *N =* 28**	***P* value**
Sex (male, *n*)	56 (58.9%)	44 (65.7%)	12 (42.9%)	0.039
Age, y, median (IQR)	72 (63–79)	72 (63–79)	74 (64–80)	0.358
**Risk factors**, ***n*** **(%)**				
Smoking	15 (15.8%)	13 (19.4%)	2 (7.1%)	0.236
Hypertension	70 (73.7%)	48 (71.6%)	22 (78.6%)	0.484
Diabetes mellitus	27 (28.4%)	18 (26.9%)	9 (32.1%)	0.603
Atrial fibrillation	23 (24.2%)	15 (22.4%)	8 (28.6%)	0.521
Baseline NIHSS score (median, IQR)	19 (16–22)	19 (15–22)	19 (18–23)	0.180
Intravenous thrombolysis, n (%)	25 (26.3%)	18 (26.9%)	7 (25.0%)	0.851
Onset-to-recanalization time, median (IQR)	349 (266–445)	323 (253–438)	385 (307–446)	0.194
ASPECT	3 (1–5)	3 (2–6)	3 (0–5)	0.158
**Occlusion site**, ***n*** **(%)**				
ICA	47 (49.5%)	32 (47.8%)	15 (56.6%)	0.606
M1 segment	39 (41.1%)	26 (38.8%)	13 (46.4%)	0.491
M2 segment	9 (9.5%)	9 (13.4%)	0 (0%)	0.098
**Endovascular therapy detail**				
Mechanical thrombectomy	93 (97.9%)	66 (98.5%)	27 (96.4%)	0.505
Angioplasty	15 (15.8%)	11 (16.4%)	4 (14.3%)	1.000
Intravenous tirofiban	29 (30.5%)	25 (37.3%)	4 (14.3%)	0.026
Intraarterial rt-PA	2 (2.1%)	1 (1.5%)	1 (3.6%)	0.505
**TOAST subtype**, ***n*** **(%)**				
CE	64 (67.4%)	40 (59.7%)	24 (85.7%)	0.014
LAA	29 (30.5%)	25 (37.3%)	4 (14.3%)	0.026
Other	2 (2.1%)	2 (3.0%)	0 (0%)	0.888
eTICI ≥ 2b, *n* (%)	94 (98.9%)	66 (98.5%)	28 (100%)	1.000
**Clinical outcome**, ***n*** **(%)**				
sICH	9 (9.5%)	3 (4.5%)	6 (21.4%)	0.029
mRS 0–2	21 (22.1%)	21 (31.3%)	0 (0%)	0.001
Mortality	32 (33.7%)	17 (25.4%)	15 (53.6%)	0.008

IQR, interquartile range; ASPECT, Alberta Stroke Program Early CT Score; TOAST, Trial of Org 10172 in Acute Stroke Treatment; NIHSS, National Institutes of Health Stroke Scale; ICA, intracranial carotid artery; LAA, large-artery atherosclerosis; CE, cardio embolism; sICH, symptomatic intracranial hemorrhage.

### Comparison between the early severe MLS presence and no early severe MLS presence groups

Table 1 shows that the TOAST subtype was different between the 2 groups. Compared with the no early severe MLS presence group, the early severe MLS presence group included more patients with the cardiac embolic type (85.7 vs. 59.7%, *P* = 0.014) and fewer with large artery atherosclerosis (LAA) (14.3 vs. 37.3%, *P* = 0.026).

The clinical outcome was poorer in the early severe MLS presence group: there were more patients with sICH (21.4 vs. 4.5%, *P* = 0.029) and fewer patients with functional independence (0 vs. 31.3%, *P* = 0.001). Furthermore, more patients died (53.6 vs. 25.4%, *P* = 0.008) in the early severe MLS presence group.

No significant differences were found between the 2 outcome groups in age (74 year in the early severe MLS presence group vs. 72 yrs. in the no early severe MLS presence group, *P* = 0.358), baseline NIHSS (19 vs. 19, *P* = 0.180), risk factors including smoking (7.1 vs. 19.4%, *P* = 0.236), hypertension (78.6 vs. 71.6%, *P* = 0.484), diabetes mellitus (32.1 vs. 26.9%, *P* = 0.603), or atrial fibrillation (28.6 vs. 22.4%, *P* = 0.521), except there were fewer male patients in the early severe MLS presence group (42.9 vs. 65.7%, *P* = 0.039). The occlusion site was similar in the 2 groups, including intracranial internal carotid artery occlusion (56.6% in the early severe MLS presence group vs. 47.8% in the no early severe MLS presence group, *P* = 0.606) and the M1 segment of the MCA (46.4 vs. 38.8%, *P* = 0.491).

No significant differences were found between the 2 outcome groups in endovascular therapy detail except more patients received intravenous tirofiban in no MLS group than that in MLS group (37.3 vs. 14.3%, *P* = 0.026).

### Baseline infarct core volume calculated by different thresholds of rCBF between the 2 groups

As Table 2 shows, compared with the no early severe MLS presence group, patients in the early severe MLS presence group showed a larger infarct core volume of rCBF < 30% (median [IQR], 91 [71–113] vs. 71 [61–93]; *P* = 0.01) and rCBF < 15% (median [IQR], 46 [32–60] vs. 29 [32–60]; *P* < 0.001). There were 29 patients with rCBF < 15% volumes > 40 ml and rCBF < 30% volumes < 130 ml, and 14 of these patients (48.3%) had early severe MLS occurrence.

**Table 2:** Comparison of baseline infarct core between patients with early severe MLS presence and without early severe MLS presence.

**Parameter**	**All patients *N =* 95**	**No MLS *N =* 67**	**MLS *N =* 28**	***P* value**
rCBF < 30%, median (IQR)	75 (64–96)	71 (61–93)	91 (71–113)	0.010
rCBF < 15%, median (IQR)	35 (23–46)	29 (19–40)	46 (32–60)	<0.001
CBF < 15% ≥40 ml, n (%)	34 (35.8%)	17 (25.4%)	17 (60.7%)	0.001

MLS, midline shift; IQR, interquartile range; ml, milliliter; rCBF, relative cerebral blood flow.

### ROC analyses and logistic regression analyses

**Figure 2:**
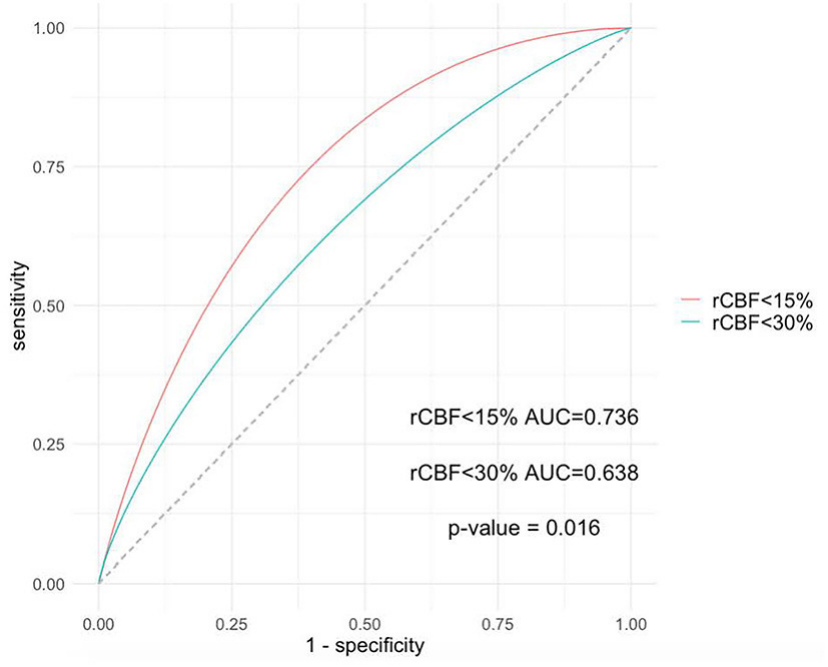
Receiver operating characteristic analyses of the rCBF < 15% volume and rCBF < 30% volume. An rCBF < 15% volume showed a better predictive value of MLS than an rCBF < 30% volume (AUC, 0.735 vs. 0.646, *P* = 0.023). rCBF indicates relative cerebral blood flow; AUC indicates the area under the curve.

Figure 2 shows that an rCBF < 15% ischaemic volume showed significantly better predictive accuracy than an ischaemic volume of rCBF < 30% (area under the curve, 0.74 vs. 0.64, *P* = 0.002). The optimal threshold points from the ROC analyses to differentiate patients likely to have a higher likelihood of MLS were core volume calculated by rCBF < 15% of ≥40 ml (sensitivity 0.61, specificity 0.76) and an rCBF < 30% core volume of ≥86 ml (sensitivity 0.61, specificity 0.70), as showed in Table 3.

**Table 3:** ROC analysis of the cut-off value of MLS.

	**AUC**	**Sensitivity**	**Specificity**	**Cut-off value**	***P* value**
rCBF < 30%	0.64 (0.52–0.75)	0.61	0.70	86	0.01
rCBF < 15%	0.74 (0.62–0.84)	0.61	0.76	40	<0.001

rCBF indicates relative cerebral blood flow.

Table 2 indicates that there were more patients with rCBF≥40 ml in the early severe MLS presence group (60.7 vs. 25.4%, *P* = 0.001). Based on the results of multivariate logistic regression, an rCBF < 15% core volume ≥ 40 ml was an independent predictor of early severe MLS presence (odds ratio [OR], 4.34 [95% CI, 1.571–11.996, *P* = 0.005]) after adjustment for sex, age, baseline NIHSS score, onset-to-recanalization time and TOAST subtype, as showed in Table 4.

**Table 4:** Multivariate logistic regression analyses of variables associated with the occurrence of MLS.

**Parameter**	**Odds ratio**	**95%CI**	***P* value**
CE	2.67	0.743–9.618	0.132
Male sex	0.40	0.137–1.160	0.091
Age, one-year increase	1.00	0.958–1.040	0.923
Onset-to-recanalization time	1.00	0.999–1.004	0.260
Baseline NIHSS score	1.00	0.923–1.154	0.579
rCBF < 15% volume ≥40 ml	4.34	1.571–11.996	0.005

CE, cardioembolism; NIHSS, National Institutes of Health Stroke Scale; rCBF, relative cerebral blood flow.

## Discussion

Our study showed that an rCBF < 15% core volume is a better tool for predicting early severe MLS presence than an rCBF < 30% core volume and an rCBF < 15% core volume ≥ 40 ml was a strong predictor of early MLS presence in patients with an LIC treated by EVT.

MLS is the gold standard imaging marker of cerebral oedema (23). MLS occurrence is one clinical scenario that patients with an LIC treated with reperfusion therapy may face and is of great concern to clinicians. MLS occurrence may be due to cerebral oedema caused by the infarction volume itself. Cerebral blood flow is the optimal CT perfusion parameter for assessing the infarct core (24), and an rCBF < 30% is traditionally defined as the infarct core (12). The chronological progression to infarction is different among different cell types and different individuals, and accurate core assessment is challenging (13). A case–control study indicated that the optimal threshold to define the ischaemic core in thrombectomy patients was an rCBF <20%, whereas in alteplase controls, the optimal ischaemic core threshold remained at an rCBF < 30% (25), and the reason may be due to earlier reperfusion. Therefore, the traditional biomarker of an rCBF < 30% ischaemic volume may also be reversible. An rCBF < 15% ischaemic volume indicates extremely ischaemic tissue, which is very difficult to reverse and save. Our study showed that compared with an rCBF < 30%, an rCBF < 15% is a better tool for predicting MLS occurrence.

In animal models, reperfusion can exacerbate cerebral oedema because severely ischaemic tissue is unlikely to survive even with reperfusion, and oedema increases after reperfusion, which supplies blood flow and water content to already infarcted tissue (21). However, clinical studies have shown that reperfusion can reduce cerebral oedema (21, 22, 26, 27). Nevertheless, reperfusion may be harmful and exacerbate cerebral oedema in patients with a very large infarct core >130 ml (21). Our study showed that an rCBF < 15% was more accurate in predicting early MLS occurrence than an rCBF < 30% and that an rCBF < 15% volume ≥ 40 ml was a strong predictor of early MLS severe occurrence, which indicated that reperfusion therapy should be used with caution in patients with an rCBF < 15% volume > 40 ml. Furthermore, our study showed that half of the patients with an rCBF < 15% volume ≥ 40 ml and an rCBF < 30% volume < 130 ml had early severe MLS occurrence. To some extent, the predictive value of rCBF < 15% volume ≥ 40 ml for the presence of MLS is better than that of rCBF < 30% volume > 130 ml.

MLS presence, especially an MLS value > 3 mm, predicts poor outcome (20, 28). In concordance, our exploratory analysis suggests that none of the patients with early severe MLS presence achieved functional independence, and half of them died. sICH is another important factor that can exacerbate cerebral oedema and cause MLS. In our study, the sICH occurrence rate was as high as 21.4%.

Our analysis adds further evidence to support the safety and benefit of EVT in patients presenting with an LIC. Our study showed that functional independence was achieved in 22.1% of patients and that sICH occurred in 9.5%, which was consistent with previous studies. Previous studies have also shown that the functional independence rate ranges from 21.7% (29) to 36.5% (11, 30, 31), the mortality rate ranges from 22.5% (31), 30.8% (31) to 44.7% (29), and the sICH occurrence rate ranges from 3.7% (32) to 7.0% (31).

The strengths of this study are the use of data from a prospective registry database, a relatively large sample and the use of objective imaging criteria with automated core volume calculations for CTP, which is more accurate than core volumes assessed with CT-ASPCET. However, our study also had some limitations.

Because of the lack of a control group that received the best medical treatment alone, we cannot judge the treatment effect sizes. Additionally, our database does not track patients with LVO who did not undergo thrombectomy, and thus, data relating to a proportion of treated patients are not available. Third, individual treatment decisions were made by different interventionalists or/and patients/families' preferences rather than on a specific selection protocol. This may have led to a selection bias that could have confounded our results but at the same time allowed us to explore the paradigm of treating patients with LIC more pragmatically. Fourth, although post-procedural neurocritical care could have some effects on the outcome, the influence may be minimized since the critical care of all these included patients was performed by a designated team. Therefore, factors regarding post-procedural critical care were not considered in the analysis.

## Conclusion

In summary, our data provide reassurance that iatrogenic exacerbation of space-occupying cerebral oedema following EVT in patients is unlikely in patients presenting with an LIC, particularly among patients with an rCBF < 15% core volume < 40 ml. However, reperfusion therapy should be used with caution in patients with an rCBF < 15% core volume ≥ 40 mL. Trials are needed to mitigate the adverse effects of cerebral oedema and offer an avenue to reduce the morbidity and mortality of patients with LIC.

## Data availability statement

The raw data supporting the conclusions of this article will be made available by the authors, without undue reservation.

## Ethics statement

The studies involving human participants were reviewed and approved by Zhangzhou Municipal Hospital Ethics Committee. The Ethics Committee waived the requirement of written informed consent for participation.

## Author contributions

All authors listed have made a substantial, direct, and intellectual contribution to the work and approved it for publication.

## References

[1] Berkhemer OA Fransen PSS Beumer D van den Berg LA Lingsma HF Yoo AJ. A randomized trial of intraarterial treatment for acute ischemic stroke. N Engl J Med. (2015) 372:11–20. 10.1056/NEJMoa1411587 25517348

[2] Goyal M Demchuk AM Menon BK Eesa M Rempel JL Thornton J. Randomized assessment of rapid endovascular treatment of ischemic stroke. N Engl J Med. (2015) 372:1019–30. 10.1056/NEJMoa1414905 25671798

[3] Saver JL Goyal M Bonafe A Diener H-C Levy EI Pereira VM. Stent-retriever thrombectomy after intravenous t-PA vs. t-PA alone in stroke N Engl J Med. (2015) 372:2285–95. 10.1056/NEJMoa1415061 25882376

[4] Campbell BC V Mitchell PJ Kleinig TJ Dewey HM Churilov L Yassi N. Endovascular therapy for ischemic stroke with perfusion-imaging selection. N Engl J Med. (2015) 372:1009–18. 10.1056/NEJMoa1414792 25671797

[5] Goyal M Menon BK van Zwam WH Dippel DWJ Mitchell PJ Demchuk AM. Endovascular thrombectomy after large-vessel ischaemic stroke: a meta-analysis of individual patient data from five randomised trials. Lancet. (2016) 387:1723–31. 10.1016/S0140-6736(16)00163-X 26898852

[6] Nogueira RG Jadhav AP Haussen DC Bonafe A Budzik RF Bhuva P. Thrombectomy 6 to 24 hours after stroke with a mismatch between deficit and infarct. N Engl J Med. (2017) 378:11–21. 10.1056/NEJMoa1706442 29129157

[7] Albers GW Marks MP Kemp S Christensen S Tsai JP Ortega-Gutierrez S. Thrombectomy for Stroke at 6 to 16 Hours with Selection by Perfusion Imaging. N Engl J Med. (2018) 378:708–18. 10.1056/NEJMoa1713973 29364767 PMC6590673

[8] Powers WJ Rabinstein AA Ackerson T Adeoye OM Bambakidis NC Becker K. Guidelines for the Early Management of Patients With Acute Ischemic Stroke: 2019 Update to the 2018 Guidelines for the Early Management of Acute Ischemic Stroke: A Guideline for Healthcare Professionals From the American Heart Association/American Stroke. Stroke. (2019) 50:e344–418. 10.1161/STR.0000000000000211 31662037

[9] Sarraj A Hassan AE Savitz S Sitton C Grotta J Chen P. Outcomes of Endovascular Thrombectomy vs Medical Management Alone in Patients with Large Ischemic Cores: A Secondary Analysis of the Optimizing Patient's Selection for Endovascular Treatment in Acute Ischemic Stroke (SELECT) Study. JAMA Neurol. (2019) 76:1147–56. 10.1001/jamaneurol.2019.2109 31355873 PMC6664381

[10] Diestro JDB Dmytriw AA Broocks G Chen K Hirsch JA Kemmling A. Endovascular thrombectomy for low aspects large vessel occlusion ischemic stroke: a systematic review and meta-analysis. Can J Neurol Sci. (2020) 47:612–9. 10.1017/cjn.2020.71 32299532

[11] Yoshimura S Sakai N Yamagami H Uchida K Beppu M Toyoda K. Endovascular therapy for acute stroke with a large ischemic region. N Engl J Med. (2022) 386:1303–13. 10.1056/NEJMoa2118191 35138767

[12] Gunasekera L Churilov L Mitchell P Bivard A Sharma G Parsons MW. Automated estimation of ischemic core prior to thrombectomy: Comparison of two current algorithms. Neuroradiology. (2021) 63:1645–9. 10.1007/s00234-021-02651-9 33580356

[13] Goyal M Ospel JM Menon B Almekhlafi M Jayaraman M Fiehler J. Challenging the ischemic core concept in acute ischemic stroke imaging. Stroke. (2020) 51:3147–3155. 10.1161/STROKEAHA.120.030620 32933417

[14] Lakomkin N Pan J Stein L Malkani B Dhamoon M Mocco J. Diffusion MRI reversibility in ischemic stroke following thrombolysis: a meta-analysis. J Neuroimaging. (2020) 30:471–6. 10.1111/jon.12703 32436311

[15] Yoo J Choi JW Lee SJ Hong JM Hong JH Kim CH. Ischemic diffusion lesion reversal after endovascular treatment: prevalence, prognosis, and predictors. Stroke. (2019) 50:1504–9. 10.1161/STROKEAHA.118.024263 31043151

[16] Adams H Adams H Bendixen B Bendixen B Kappelle L Kappelle L. Classification of subtype of acute ischemic stroke. Stroke. (1993) 23:35–41. 10.1161/01.STR.24.1.35 7678184

[17] Von Kummer R Broderick JP Campbell BCV Demchuk A Goyal M Hill MD. The heidelberg bleeding classification: Classification of bleeding events after ischemic stroke and reperfusion therapy. Stroke. (2015) 46:2981–6. 10.1161/STROKEAHA.115.010049 26330447

[18] Lin L Bivard A Parsons MW. Perfusion patterns of ischemic stroke on computed tomography perfusion. J Stroke. (2013) 15:164. 10.5853/jos.2013.15.3.164 24396810 PMC3859000

[19] Liebeskind DS Bracard S Guillemin F Jahan R Jovin TG Majoie CBLM. ETICI reperfusion: Defining success in endovascular stroke therapy. J Neurointerv Surg. (2019) 11:433–8. 10.1136/neurintsurg-2018-014127 30194109

[20] McKeown ME Prasad A Kobsa J Top I Snider SB Kidwell C. Midline shift greater than 3 mm independently predicts outcome after ischemic stroke. Neurocrit Care. (2022) 36:46–51. 10.1007/s12028-021-01341-x 34494212 PMC8813904

[21] Ng FC Yassi N Sharma G Brown SB Goyal M Majoie CBLM. Cerebral edema in patients with large hemispheric infarct undergoing reperfusion treatment: A hermes meta-analysis. Stroke. (2021) 52:3450–3458. 10.1161/STROKEAHA.120.033246 34384229 PMC8545835

[22] Kimberly WT Dutra BG Boers AMM Alves HCBR Berkhemer OA Van Den Berg L. Association of reperfusion with brain edema in patients with acute ischemic stroke: A secondary analysis of the MR CLEAN Trial. JAMA Neurol. (2018) 75:453–61. 10.1001/jamaneurol.2017.5162 29365017 PMC5885187

[23] Battey TWK Karki M Singhal AB Wu O Sadaghiani S Campbell BCV. Brain edema predicts outcome after nonlacunar ischemic stroke. Stroke. (2014) 45:3643–8. 10.1161/STROKEAHA.114.006884 25336512 PMC4295905

[24] Campbell BCV Christensen S Levi CR Desmond PM Donnan GA Davis SM. Cerebral blood flow is the optimal CT perfusion parameter for assessing infarct core. Stroke. (2011) 42:3435–40. 10.1161/STROKEAHA.111.618355 21980202

[25] Bivard A Kleinig T Miteff F Butcher K Lin L Levi C. Ischemic core thresholds change with time to reperfusion: A case control study. Ann Neurol. (2017) 82:995–1003. 10.1002/ana.25109 29205466 PMC6712948

[26] Thorén M Dixit A Escudero-Martínez I Gdovinová Z Klecka L Rand VM. Effect of recanalization on cerebral edema in ischemic stroke treated with thrombolysis and/or endovascular therapy. Stroke. (2020) 51:216–23. 10.1161/STROKEAHA.119.026692 31818228

[27] Jo KW Bajgur SS Kim H Choi HA Huh PW Lee K. simple prediction score system for malignant brain edema progression in large hemispheric infarction. PLoS ONE. (2017) 12:1–12. 10.1371/journal.pone.0171425 28178299 PMC5298259

[28] Kim H Tak S Woo Y Rim S Seong I Wook K. Predictors of malignant brain edema in middle cerebral artery infarction observed on CT angiography. J Clin Neurosci. (2014) 22:554–60. 10.1016/j.jocn.2014.08.021 25510537

[29] Deb-Chatterji M Pinnschmidt H Flottmann F Leischner H Broocks G Alegiani A. Predictors of independent outcome of thrombectomy in stroke patients with large baseline infarcts in clinical practice: A multicenter analysis. J Neurointerv Surg. (2020) 12:1064–8. 10.1136/neurintsurg-2019-015641 32107288

[30] Bouslama M Barreira CM Haussen DC Rodrigues GM Pisani L Frankel MR. Endovascular reperfusion outcomes in patients with a stroke and low ASPECTS is highly dependent on baseline infarct volumes. J Neurointerv Surg. (2021) 14:117–21. 10.1136/neurintsurg-2020-017184 33722970

[31] Zaidat OO Liebeskind DS Jadhav AP Ortega-Gutierrez S Nguyen TN Haussen DC. Impact of age and alberta stroke program early computed tomography score 0 to 5 on mechanical thrombectomy outcomes: analysis from the STRATIS registry. Stroke. (2021) 52:2220–8. 10.1161/STROKEAHA.120.032430 34078106 PMC8240495

[32] Kakita H Yoshimura S Uchida K Sakai N Yamagami H Morimoto T. Impact of endovascular therapy in patients with large ischemic core: subanalysis of recovery by endovascular salvage for cerebral ultra-acute embolism Japan registry 2. Stroke. (2019) 50:901–8. 10.1136/neurintsurg-2018-SNIS.224 31633899

